# Atrial fibrillation as an independent risk factor for venous thromboembolism in intracerebral hemorrhage patients: a multicenter retrospective cohort study

**DOI:** 10.3389/fneur.2026.1796208

**Published:** 2026-05-22

**Authors:** Hongfu Chen, Jianyong Ji, Hui Zhang, Er Nie, Qing Lan

**Affiliations:** 1 Department of Neurosurgery, The Second Affiliated Hospital of Soochow University, Suzhou, Jiangsu, China; 2 Department of Neurosurgery, The Affiliated Hospital of Xuzhou Medical University, Xuzhou, Jiangsu, China; 3 Department of Neurosurgery, Liaocheng People’s Hospital, Liaocheng, Shandong, China

**Keywords:** atrial fibrillation, intracerebral hemorrhage, risk factors, thromboprophylaxis, venous thromboembolism

## Abstract

**Background:**

Venous thromboembolism (VTE) represents a major and potentially fatal complication among patients with intracerebral hemorrhage (ICH); however, accurate risk stratification remains difficult in this population. The present study sought to determine independent predictors of VTE in patients with ICH using data derived from a large multicenter critical care database.

**Methods:**

A retrospective cohort study was performed using the eICU Collaborative Research Database. Adult patients with a diagnosis of ICH were included. VTE events were identified through an integrated multi-modal approach incorporating diagnostic coding, clinical text mining, and records of therapeutic anticoagulation. Data on baseline demographics, comorbid conditions, laboratory parameters, and thromboprophylaxis practices were collected. Univariate and multivariate logistic regression models were applied to identify independent risk factors for VTE. Sensitivity analyses were conducted using alternative VTE definitions to assess the robustness of the findings. Interaction analyses and additional sensitivity analyses excluding anticoagulation-only VTE cases were performed to further evaluate the robustness of the primary findings.

**Results:**

A total of 785 patients with ICH were analyzed, of whom 53 (6.75%) developed VTE during hospitalization. In univariate analyses, nine variables were significantly associated with VTE occurrence, including atrial fibrillation (AF) (OR = 5.75, 95% CI: 2.95–11.21, *p* < 0.001), chronic obstructive pulmonary disease (COPD) (OR = 6.81, 95% CI: 2.67–17.38, *p* < 0.001), and use of mechanical ventilation (OR = 2.53, 95% CI: 1.38–4.64, *p* = 0.003). After adjustment for potential confounders in multivariate analysis, atrial fibrillation emerged as the only independent risk factor for VTE (adjusted OR = 3.84, 95% CI: 1.75–8.41, *p* < 0.001). The incidence of VTE was substantially higher among patients with AF compared with those without AF, representing an unadjusted 4.6-fold higher VTE incidence (24.2% vs. 5.3%). This association remained consistent across multiple sensitivity analyses, including analyses excluding anticoagulation-only VTE cases (adjusted OR = 3.99, 95% CI: 1.02–15.55, *p* = 0.046).

**Conclusion:**

Atrial fibrillation was the only independent risk factor for VTE after multivariable adjustment, conferring an approximately four-fold increase in VTE risk, and should be interpreted as a clinical risk marker rather than a direct causal factor. Patients with ICH complicated by AF constitute a particularly high-risk subgroup and may benefit from intensified surveillance and individualized thromboprophylaxis strategies.

## Introduction

### Background

Intracerebral hemorrhage (ICH) accounts for approximately 10–15% of all stroke cases and is associated with substantial mortality and long-term disability (1, 2). Although ICH is primarily a hemorrhagic disorder, patients paradoxically face an increased risk of thromboembolic complications, particularly venous thromboembolism (VTE), which includes deep vein thrombosis (DVT) and pulmonary embolism (PE) (3, 4). Previous studies have reported that VTE occurs in 1–10% of patients with ICH and is associated with a markedly increased risk of mortality (5, 6).

### Clinical dilemma

Prevention of VTE in patients with ICH represents a distinct clinical challenge. Although pharmacological thromboprophylaxis has been shown to effectively reduce the risk of VTE in general intensive care unit populations, its use in patients with ICH remains controversial because of concerns regarding hematoma expansion (7, 8). This dilemma is further intensified in patients with concomitant atrial fibrillation (AF), who typically require long-term anticoagulation for ischemic stroke prevention but are generally considered to have absolute contraindications to anticoagulation during the acute phase following ICH (9).

### Knowledge gaps

Current evidence regarding risk factors for VTE in patients with ICH remains incomplete. Prior studies have reported potential associations with factors such as immobility, lower extremity paresis, advanced age, and the use of mechanical ventilation (10, 11). However, most available data are derived from small, single-center studies and are limited by the absence of comprehensive multivariate analyses. Moreover, despite the high prevalence of AF among patients with ICH, its specific contribution to VTE risk in this population has not been consistently identified as an independent risk factor, likely reflecting differences in sample size, AF prevalence, and statistical adjustment strategies (12). Therefore, this multicenter retrospective cohort study was conducted to identify independent risk factors for VTE in patients with ICH, with a particular focus on the contribution of AF.

## Methods

### Study design and data source

This study was conducted as a retrospective cohort analysis based on the eICU Collaborative Research Database (eICU-CRD), a large multicenter intensive care unit database that contains de-identified clinical information from more than 200,000 ICU admissions across 208 hospitals in the United States (13). The eICU-CRD (Version 2.0) contains data collected during 2014–2015. Access to the database was obtained through the PhysioNet platform after completion of the required training and data-use approval process.

### Study population

We included adult patients (≥18 years) with a primary diagnosis of ICH, identified using International Classification of Diseases, Ninth Revision (ICD-9) codes, who were admitted to an ICU within the eICU Collaborative Research Database. As the eICU database captures ICU-level encounters, the study population represents patients with acute ICH requiring intensive care management.

### Inclusion criteria

Adult patients (aged ≥18 years) admitted to the intensive care unit with a primary or secondary diagnosis of ICH.

### Exclusion criteria

Patients were excluded based on the following criteria: (1) missing APACHE IV score (*n* = 1,253), which requires complete physiological data within the first 24 h of ICU admission-missing scores predominantly reflect transfers, very short stays, or technical issues with data capture; (2) missing age or sex (*n* = 584), likely due to de-identification procedures or data entry gaps; (3) data quality issues including logical inconsistencies such as negative length-of-stay values, impossible vital sign combinations, or duplicate records (*n* = 356). The final analytic cohort comprised 785 patients (Supplementary Table 3).

### AF identification

Atrial fibrillation (AF) was identified from the patient medical history and admission diagnosis tables within the eICU database, using structured data fields (APACHE comorbidity entries). This approach captures AF documented as a pre-existing comorbidity at the time of ICU admission, rather than new-onset AF during ICU stay. Therefore, the AF variable in our analysis predominantly represents pre-existing (chronic) AF, which is clinically relevant as it reflects the patient’s baseline prothrombotic risk profile and prior anticoagulation exposure.

### VTE identification strategy

Venous thromboembolism (VTE) events were identified using a multi-source evidence integration approach designed to maximize sensitivity while preserving diagnostic specificity.

#### Diagnostic evidence

##### ICD-9 diagnostic codes

Deep vein thrombosis (DVT): 451.1x (phlebitis and thrombophlebitis of deep vessels), 453.4x (venous embolism and thrombosis of deep vessels).

Pulmonary embolism (PE): 415.1x (pulmonary embolism and infarction).

##### Text mining of diagnostic narratives

DVT-related patterns: “deep vein thromb”, “DVT”, “lower extremity thromb”.

PE-related patterns: “pulmonary embol” (exact word-boundary matching was applied to minimize false-positive matches).

#### Therapeutic evidence

Therapeutic anticoagulation was considered supportive evidence for VTE when administered at treatment doses, excluding prophylactic regimens. Eligible anticoagulant therapies included.

Warfarin or other vitamin K antagonists
Therapeutic-dose low molecular weight heparin (LMWH)
Continuous intravenous unfractionated heparin infusion
Direct oral anticoagulants (DOACs)

#### Confidence stratification of VTE events

VTE events were categorized according to the level of diagnostic certainty.

- *High confidence*: presence of both diagnostic evidence and therapeutic anticoagulation.
- *Medium confidence*: presence of diagnostic evidence or therapeutic anticoagulation alone.
- *Primary definition*: high-and medium-confidence VTE events.
- *Sensitivity analysis*: all identified VTE events regardless of confidence level.

### Variable extraction

*Demographics variables*: age, Sex.

*Disease severity indicators*: acute Physiology and Chronic Health Evaluation IV (APACHE IV) score, Glasgow Coma Scale (GCS) score, Requirement for mechanical ventilation.

*Comorbidities*: comorbid conditions were identified using a combination of ICD-9 diagnostic codes and diagnosis-related text, including: Hypertension, Diabetes mellitus, Atrial fibrillation, Chronic kidney disease, Congestive heart failure, Chronic obstructive pulmonary disease (COPD).

*Laboratory variables*: the first available laboratory values recorded during the ICU stay were extracted, including: Complete blood count (white blood cell count, hemoglobin, platelet count), Serum creatinine, Blood glucose, Coagulation parameters (prothrombin time, international normalized ratio, activated partial thromboplastin time).

*Thromboprophylaxis measures*: use of sequential compression devices (SCDs), Use of compression stockings.

*Clinical outcomes:* ICU length of stay and hospital length of stay, ICU mortality and in-hospital mortality.

### Statistical analysis

#### Descriptive statistics

Continuous variables were assessed for normality using the Shapiro–Wilk test. Variables following a normal distribution are presented as mean ± standard deviation (SD); non-normally distributed variables are presented as median (interquartile range [IQR]). Continuous variables were compared using independent-samples t-tests or Mann–Whitney U tests as appropriate. Categorical variables are reported as counts (percentages) and were compared using the chi-square test or Fisher’s exact test when the expected cell count was <5. All statistical tests were two-tailed, and statistical significance was set at *p* < 0.05.

#### Univariate analysis

Univariate logistic regression analyses were performed to evaluate the association between each potential risk factor and VTE. Unadjusted odds ratios (ORs) with corresponding 95% confidence intervals (CIs) were calculated.

#### Multivariable analysis

Variables were selected for inclusion in the multivariable logistic regression model based on the following criteria:

1. Statistical significance in univariate analysis (*p* < 0.01).
2. Established clinical relevance.
3. An events-per-variable (EPV) ratio ≥10 to ensure model stability.

The final multivariable model included five variables: atrial fibrillation, chronic obstructive pulmonary disease, mechanical ventilation, use of sequential compression devices, and Glasgow Coma Scale score. Multicollinearity was assessed using variance inflation factors (VIFs). Model performance and goodness-of-fit were evaluated using pseudo-*R*^2^, Akaike information criterion (AIC), and Bayesian information criterion (BIC).

#### Sensitivity analysis

To assess the robustness of the results, multivariable logistic regression analyses were repeated using alternative definitions of VTE, including high-confidence VTE events only, the recommended definition (high- and medium-confidence events), and all identified VTE events. An additional sensitivity analysis was performed excluding VTE cases identified solely through therapeutic anticoagulation records (anticoagulation-only VTE), retaining only cases with diagnostic evidence (ICD-9 codes or text-mining identification), to address concerns about potential outcome misclassification in AF patients.

### Interaction analysis

Exploratory interaction analyses were performed to evaluate potential effect modification between AF and other covariates (mechanical ventilation, COPD) on VTE risk. Interaction terms were added to the multivariable logistic regression model, and their significance was assessed using the Wald test and likelihood ratio test.

### Statistical software

All statistical analyses were conducted using Python (version 3.13). Data processing was performed with pandas, logistic regression analyses were carried out using statsmodels, statistical tests were conducted with scipy, and data visualization was generated using matplotlib.

### Ethical considerations

This study was conducted using de-identified data obtained from the eICU Collaborative Research Database (eICU-CRD). The original data collection was approved by the institutional review boards of the participating hospitals, with a waiver of informed consent. The present study was deemed exempt from additional institutional review board review, as it involved secondary analysis of publicly available, de-identified data.

## Results

### Study population

**Figure 1:**
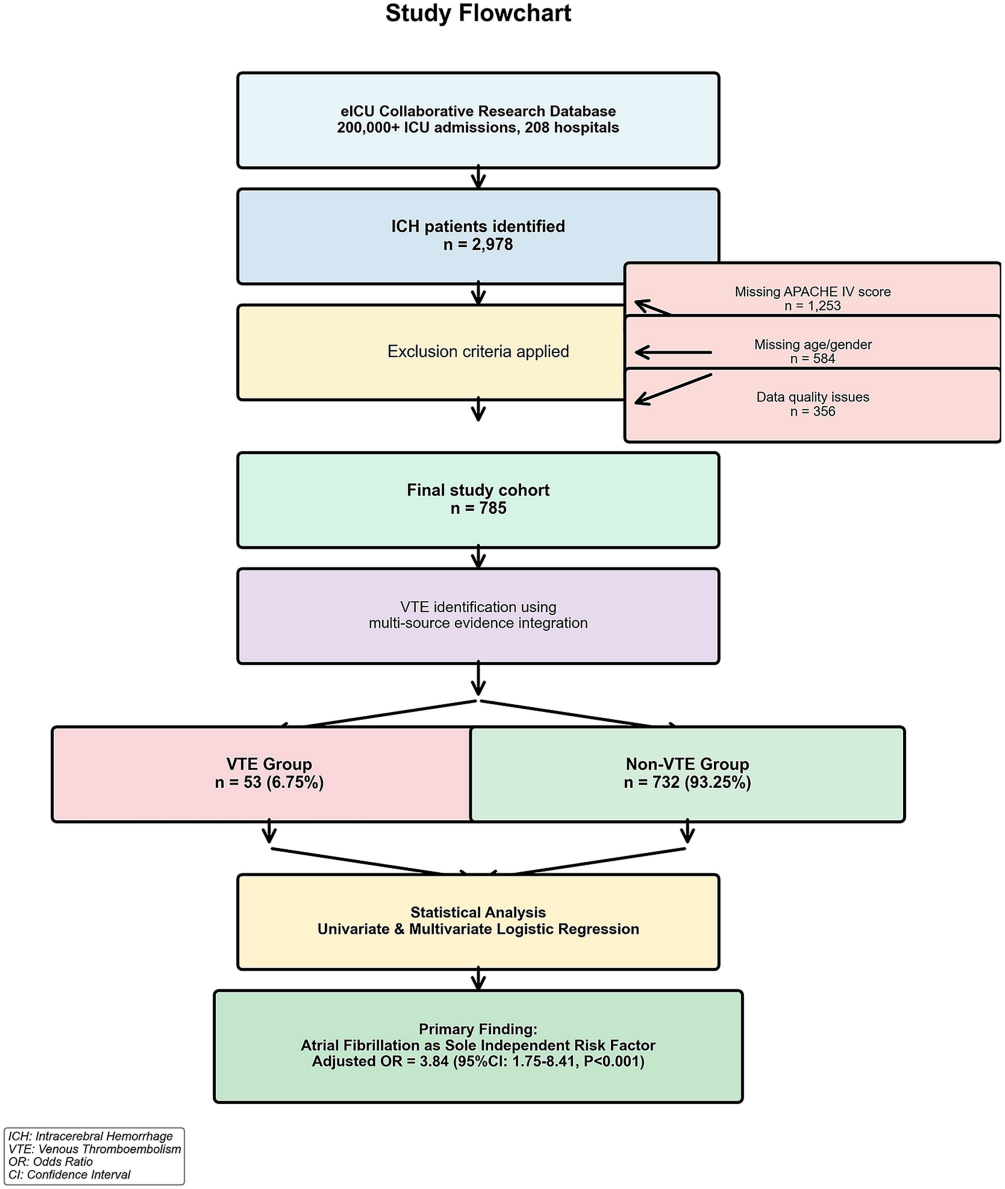
Study flowchart study flowchart illustrating patient selection and cohort derivation. From the eICU collaborative research database (2014–2015), which contains over 200,000 ICU admissions from 208 hospitals, 2,978 patients with ICH were initially identified. After applying exclusion criteria, including missing APACHE IV scores (*n* = 1,253), missing age or sex (*n* = 584), and data quality issues (*n* = 356), a total of 785 patients were included in the final analysis. VTE was identified using a multi-source evidence integration approach, yielding 53 VTE cases (6.75%) and 732 non-VTE cases (93.25%). ICH, intracerebral hemorrhage; VTE, venous thromboembolism; OR, odds ratio; CI, confidence interval.

A total of 2,978 patients with ICH were initially identified from the eICU Collaborative Research Database. After applying exclusion criteria-missing APACHE IV scores (*n* = 1,253), missing age or sex (*n* = 584), and data quality issues (*n* = 356) — 785 patients were included in the final analysis (Figure 1). The mean age of the study population was 60.2 ± 20.2 years, and 304 patients (38.7%) were male. A comparison of included versus excluded patients on available variables is presented in Supplementary Table 3.

### VTE incidence

Using the recommended definition incorporating high- and medium-confidence events, 53 patients (6.75%) developed VTE during their ICU stay. Among these, 4 patients (0.51%) met criteria for high-confidence VTE, while 49 patients (6.24%) were classified as medium-confidence VTE. When all confidence levels were considered, including low-confidence events, the overall incidence of VTE remained 6.75%.

With respect to VTE subtype, 28 patients (3.57%) developed deep vein thrombosis (DVT) alone, 5 patients (0.64%) developed pulmonary embolism (PE) alone, and 3 patients (0.38%) experienced both DVT and PE. In addition, 17 patients (2.17%) received therapeutic anticoagulation in the absence of a documented diagnostic code for VTE.

### Baseline characteristics

Baseline characteristics stratified by VTE status are summarized in Table 1. All continuous variables were non-normally distributed (Shapiro–Wilk test, all *p* < 0.001); therefore, they are presented as median (IQR) and compared using Mann–Whitney *U* tests. Hemoglobin values were corrected by removing implausible outliers (>30 g/dL, *n* = 4 values removed).

**Table 1:** Baseline characteristics of ICH patients by VTE status [REVISED].

Characteristic	VTE group (*n* = 53)	Non-VTE group (*n* = 732)	*p* value
Demographics			
Age, years, median (IQR)	61.0 (43.0–71.0)	63.0 (48.0–77.0)	0.072
Male sex, *n* (%)	12 (22.6%)	292 (39.9%)	0.019*
Disease severity			
APACHE IV score, median (IQR)	57.5 (42.2–79.8)	50.0 (36.0–71.0)	0.081
GCS score, median (IQR)	8.0 (5.0–14.0)	13.0 (7.0–15.0)	0.007**
Predicted ICU mortality, median (IQR)	0.1 (0.0–0.2)	0.1 (0.0–0.2)	0.321
Mechanical ventilation, *n* (%)	23 (43.4%)	199 (27.2%)	0.004**
Comorbidities			
Hypertension, *n* (%)	14 (26.4%)	155 (21.2%)	0.470
Diabetes mellitus, *n* (%)	1 (1.9%)	7 (1.0%)	0.430
Atrial fibrillation, *n* (%)	15 (28.3%)	47 (6.4%)	<0.001***
Chronic kidney disease, *n* (%)	1 (1.9%)	27 (3.7%)	1.000
Congestive heart failure, *n* (%)	2 (3.8%)	8 (1.1%)	0.142
COPD, *n* (%)	7 (13.2%)	16 (2.2%)	<0.001***
Laboratory values			
WBC, ×10^9^/L, median (IQR)	11.1 (7.7–15.0)	10.3 (7.6–14.0)	0.368
Hemoglobin, g/dL, median (IQR)^a^	11.9 (10.1–13.7)	12.9 (11.3–14.2)	0.017*
Platelets, ×10^9^/L, median (IQR)	219.0 (163.0–281.0)	213.0 (167.0–263.0)	0.945
Creatinine, mg/dL, median (IQR)	1.0 (0.8–1.1)	0.9 (0.7–1.1)	0.097
Glucose, mg/dL, median (IQR)	129.0 (98.0–171.0)	126.0 (105.0–162.2)	0.692
PT, seconds, median (IQR)	14.4 (1.4–24.2)	15.4 (1.5–27.0)	0.442
INR, median (IQR)	1.2 (1.1–1.4)	1.1 (1.0–1.2)	0.003**
PTT, seconds, median (IQR)	28.0 (25.3–34.8)	28.0 (25.0–32.0)	0.340
Prophylaxis measures			
SCD use, *n* (%)	39 (73.6%)	641 (87.6%)	0.007**
Compression stockings, *n* (%)	0 (0%)	0 (0%)	—
Outcomes			
ICU LOS, days, median (IQR)	5.2 (1.9–9.3)	2.0 (1.0–4.3)	<0.001***
Hospital LOS, days, median (IQR)	11.9 (7.0–20.2)	5.6 (2.7–10.7)	<0.001***
ICU mortality, *n* (%)	4 (7.5%)	78 (10.7%)	0.637

VTE, venous thromboembolism; IQR, interquartile range; APACHE, Acute Physiology and Chronic Health Evaluation; GCS, Glasgow Coma Scale; ICU, intensive care unit; COPD, chronic obstructive pulmonary disease; WBC, white blood cell count; PT, prothrombin time; INR, international normalized ratio; PTT, partial thromboplastin time; SCD, sequential compression device; LOS, length of stay. *p < 0.05; **p < 0.01; ***p < 0.001.

a Hemoglobin values corrected by removing implausible outliers (>30 g/dL, *n* = 4). All continuous variables were non-normally distributed (Shapiro–Wilk test, all *p* < 0.001) and are presented as median (IQR) with Mann–Whitney *U* tests.

Significant differences between patients with and without VTE (*p* < 0.05) were observed across several domains. In terms of demographics, female sex was more frequent among patients who developed VTE (77.4% vs. 60.1%, *p* = 0.019). Regarding disease severity, patients with VTE had lower GCS scores (median 8.0 [IQR 5.0–14.0] vs. 13.0 [7.0–15.0], *p* = 0.007) and a higher prevalence of mechanical ventilation (43.4% vs. 27.2%, *p* = 0.004).

With respect to comorbidities, AF was substantially more common in the VTE group (15 of 53 [28.3%] vs. 47 of 732 [6.4%], *p* < 0.001), representing the most pronounced between-group difference. Conversely, VTE incidence was markedly higher among AF patients (15 of 62 [24.2%]) than among non-AF patients (38 of 723 [5.3%]). COPD was also more frequent among patients with VTE (13.2% vs. 2.2%, p < 0.001).

Hemoglobin levels, after removal of outliers, were lower in the VTE group (median 11.9 [IQR 10.1–13.7] vs. 12.9 [11.3–14.2] g/dL, *p* = 0.017). INR was slightly higher in the VTE group (median 1.2 [IQR 1.1–1.4] vs. 1.1 [1.0–1.2], *p* = 0.003).

Differences were additionally observed in thromboprophylaxis measures and clinical outcomes. Use of SCDs was lower in patients with VTE (73.6% vs. 87.6%, *p* = 0.007). Patients who developed VTE experienced longer ICU stays (median 5.2 [IQR 1.9–9.3] vs. 2.0 [1.0–4.3] days, *p* < 0.001) as well as longer overall hospital stays (median 11.9 [IQR 7.0–20.2] vs. 5.6 [2.7–10.7] days, *p* < 0.001).

No statistically significant differences were observed between groups with respect to age, APACHE IV score, hypertension, diabetes mellitus, or most other laboratory parameters.

### Univariate analysis

The results of the univariate logistic regression analysis are summarized in Table 2. Nine variables were significantly associated with VTE (*p* < 0.05).

**Table 2:** Univariate logistic regression analysis of risk factors for VTE.

Variable	OR	95% CI	*p* value	Significance
Strongest risk factors				
COPD	6.81	2.67–17.38	<0.001	
Atrial fibrillation	5.75	2.95–11.21	<0.001	
Hospital LOS (per day)	1.05	1.03–1.08	<0.001	
PTT (per second)	1.03	1.01–1.06	0.002	
Mechanical ventilation	2.53	1.38–4.64	0.003	
Protective factors				
SCD use	0.40	0.21–0.76	0.005	
GCS score (per point)	0.93	0.87–0.98	0.008	
Male sex	0.50	0.26–0.96	0.037	*
Other risk factors				
ICU LOS (per day)	1.03	1.00–1.06	0.045	*
Non-significant factors				
Age (per year)	0.99	0.97–1.00	0.095	NS
APACHE IV score (per point)	1.01	1.00–1.02	0.154	NS
Congestive heart failure	3.55	0.73–17.15	0.115	NS
INR (per unit)	1.16	0.87–1.54	0.323	NS
WBC (per ×10^9^/L)	1.02	0.98–1.06	0.356	NS
Hypertension	1.34	0.71–2.52	0.372	NS
Chronic kidney disease	0.50	0.07–3.77	0.503	NS
PT (per second)	0.99	0.98–1.01	0.508	NS
Diabetes mellitus	1.99	0.24–16.50	0.523	NS
Hemoglobin (per g/dL)	1.01	0.98–1.04	0.552	NS
Creatinine (per mg/dL)	0.95	0.71–1.26	0.708	NS
Platelets (per ×10^9^/L)	1.00	1.00–1.00	0.977	NS
Glucose (per mg/dL)	1.00	1.00–1.00	0.978	NS

OR, odds ratio; CI, confidence interval; NS, not significant; GCS, Glasgow Coma Scale; APACHE, Acute Physiology and Chronic Health Evaluation; ICU, intensive care unit; LOS, length of stay; COPD, chronic obstructive pulmonary disease; WBC, white blood cell count; PT, prothrombin time; INR, international normalized ratio; PTT, partial thromboplastin time; SCD, sequential compression device. *p < 0.05; **p < 0.01; ***p < 0.001.

Among the strongest risk factors, COPD demonstrated the highest odds ratio (OR = 6.81, 95% CI: 2.67–17.38, *p* < 0.001), followed by AF (OR = 5.75, 95% CI: 2.95–11.21, *p* < 0.001) and mechanical ventilation (OR = 2.53, 95% CI: 1.38–4.64, *p* = 0.003). Coagulation and exposure-related variables were also significant, including prolonged activated partial thromboplastin time (PTT; OR = 1.03 per unit increase, 95% CI: 1.01–1.06, *p* = 0.002), longer hospital length of stay (OR = 1.05 per day, 95% CI: 1.03–1.08, *p* < 0.001), and longer ICU length of stay (OR = 1.03 per day, 95% CI: 1.00–1.06, *p* = 0.045).

Several variables exhibited protective associations with VTE. Use of SCDs was associated with a lower risk of VTE (OR = 0.40, 95% CI: 0.21–0.76, *p* = 0.005). Higher GCS scores were inversely associated with VTE risk (OR = 0.93 per point increase, 95% CI: 0.87–0.98, *p* = 0.008), as was male sex (OR = 0.50, 95% CI: 0.26–0.96, *p* = 0.037).

### Multivariable analysis

The results of the multivariable logistic regression analysis are presented in Table 3. The final model included five variables selected on the basis of statistical significance in univariate analyses and clinical relevance. The model demonstrated an acceptable fit, with a pseudo-*R*^2^ of 0.0773 and an AIC of 323.24. The events-per-variable ratio was adequate (45 events across 5 variables; EPV = 9.0).

**Table 3:** Multivariable logistic regression analysis of independent risk factors for VTE [REVISED].

Variable	Unadjusted OR	Unadjusted 95% CI	Adjusted OR	Adjusted 95% CI	*p* value	OR change
Atrial fibrillation	5.75	2.95–11.21	3.84	1.75–8.41	<0.001***	−33%
COPD	6.81	2.67–17.38	2.30	0.56–9.48	0.250	−66%
Mechanical ventilation	2.53	1.38–4.64	2.00	0.83–4.79	0.122	−21%
SCD use	0.40	0.21–0.76	0.47	0.22–1.03	0.060	+20%
GCS score (per point)	0.93	0.87–0.98	0.97	0.89–1.05	0.460	+5%

Complete case analysis: 725 patients, 45 VTE events (EPV = 9.0). Model fit: pseudo-*R*^2^ = 0.0773, AIC = 323.24, BIC = 350.75. The OR change column shows the percentage change from unadjusted to adjusted OR. After adjusting for confounders, only AF remained an independent risk factor for VTE, showing the least attenuation (−33%).

OR, odds ratio; CI, confidence interval; COPD, chronic obstructive pulmonary disease; SCD, sequential compression device; GCS, Glasgow Coma Scale; AIC, Akaike information criterion; BIC, Bayesian information criterion; EPV, events per variable. ***p < 0.001.

After adjustment for covariates, AF remained the only variable independently associated with VTE (adjusted OR = 3.84, 95% CI: 1.75–8.41, *p* < 0.001).

**Figure 2:**
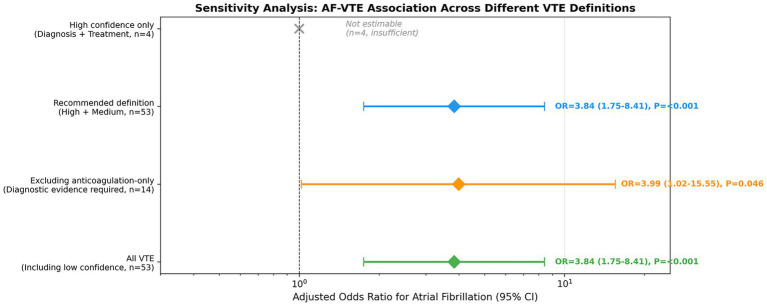
Forest plot: Univariate vs. multivariate analysis Forest plot comparing unadjusted (univariate) and adjusted (multivariate) odds ratios for VTE risk factors in patients with ICH. Five variables were included in the multivariable model: AF, COPD, mechanical ventilation, SCD use, and GCS score. Blue diamonds represent unadjusted odds ratios; pink diamonds represent adjusted odds ratios. Error bars indicate 95% confidence intervals. Specific OR values, 95% CIs, and *p* values are annotated for each variable. The vertical dashed line denotes an odds ratio of 1.0 (no association). After multivariable adjustment, AF remained the only variable significantly associated with VTE (adjusted OR = 3.84, 95% CI: 1.75–8.41, *p* < 0.001). COPD, chronic obstructive pulmonary disease; SCD, sequential compression device; GCS, Glasgow Coma Scale; OR, odds ratio; CI, confidence interval.

Several variables that were significant in univariate analyses did not retain statistical significance after multivariable adjustment. COPD was no longer significantly associated with VTE (adjusted OR = 2.30, 95% CI: 0.56–9.48, *p* = 0.250), with a marked attenuation of effect size compared with the unadjusted estimate. Similarly, mechanical ventilation did not remain significant after adjustment (adjusted OR = 2.00, 95% CI: 0.83–4.79, *p* = 0.122). The GCS score was also not independently associated with VTE in the adjusted model (adjusted OR = 0.97, 95% CI: 0.89–1.05, *p* = 0.460). Use of SCDs demonstrated a borderline protective association with VTE that did not reach conventional statistical significance (adjusted OR = 0.47, 95% CI: 0.22–1.03, *p* = 0.060). Figure 2 presents the univariate and multivariate regression results in forest plot format for visual comparison of effect estimates.

### VTE incidence by stratified AF status

Among the 62 patients with AF, VTE developed in 15 patients, corresponding to an incidence of 24.2%. In contrast, among the 723 patients without AF, 38 patients (5.3%) experienced VTE. This translated into an unadjusted 4.6-fold higher VTE incidence among patients with AF compared with those without AF (24.2% vs. 5.3%). The absolute risk difference was 18.9%, corresponding to a number needed to harm of approximately 5. These findings indicate that, within this cohort, one additional VTE event occurred for approximately every five patients with AF compared with patients without AF.

### Sensitivity analysis

**Figure 3:**
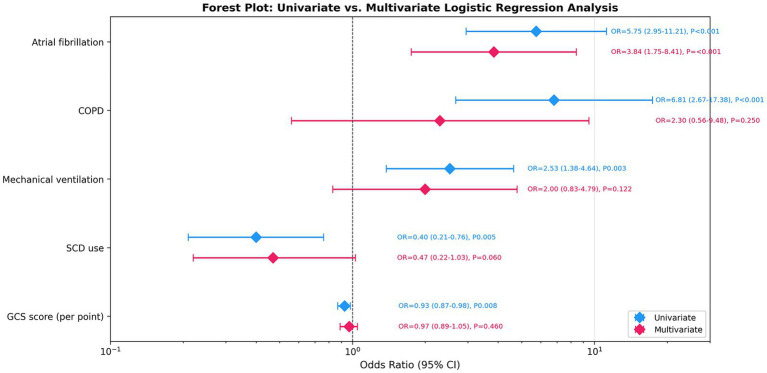
Sensitivity analysis: AF-VTE association across different VTE definitions forest plot presenting sensitivity analysis results for the AF-VTE association across different VTE definitions. Four definitions were compared: high confidence only (*n* = 4, insufficient for multivariate analysis), recommended definition (high + medium confidence, *n* = 53), excluding anticoagulation-only VTE (*n* = 14), and all VTE (*n* = 53). Error bars represent 95% confidence intervals, and the vertical dashed line denotes an odds ratio of 1.0. Specific OR values, 95% CIs, and *p* values are annotated. The AF-VTE association remained significant across all analyzable definitions, including the sensitivity analysis excluding anticoagulation-only cases (adjusted OR = 3.99, 95% CI: 1.02–15.55, *p* = 0.046). VTE, venous thromboembolism; AF, atrial fibrillation; OR, odds ratio; CI, confidence interval.

Sensitivity analysis results across different definitions of VTE are summarized in Table 4 and illustrated in Figure 3. The association between AF and VTE remained consistent across all analyzed definitions, with stable adjusted odds ratios, confidence intervals, and *p* values, indicating robustness of the primary finding.

**Table 4:** Sensitivity analysis: association between atrial fibrillation and VTE across different VTE definitions [REVISED].

VTE definition	VTE events (*n*)	AF crude OR	AF adjusted OR (95% CI)	*p* value
Primary (high + medium confidence)	53	5.75	3.84 (1.75–8.41)	<0.001***
Excluding anticoagulation-only cases^a^	14	4.92	3.99 (1.02–15.55)	0.046*
High confidence only (diagnosis + treatment)	4	36.71	Not estimable^b^	—
Diagnosis-based only (no anticoag evidence)^c^	10	1.30	Not estimable^b^	—

Adjusted OR derived from multivariable logistic regression adjusted for COPD, mechanical ventilation, SCD use, and GCS score.

VTE, venous thromboembolism; AF, atrial fibrillation; OR, odds ratio; CI, confidence interval; COPD, chronic obstructive pulmonary disease; SCD, sequential compression device; GCS, Glasgow Coma Scale. *p < 0.05; ***p < 0.001.

a Retained VTE cases with diagnostic evidence (ICD-9 codes or clinical text mining), excluding 39 cases identified solely through therapeutic anticoagulation records. Among the 43 VTE cases with therapeutic anticoagulation, 29 (67.4%) occurred in non-AF patients.

b Insufficient events for reliable multivariate analysis (minimum 10 events per variable required).

c Medium-confidence VTE cases identified through diagnostic evidence without therapeutic anticoagulation; excludes high-confidence cases.

Critically, when VTE cases identified solely through therapeutic anticoagulation records were excluded (retaining only cases with diagnostic evidence, *n* = 14 VTE events), the AF–VTE association remained significant (adjusted OR = 3.99, 95% CI: 1.02–15.55, *p* = 0.046). Among the 43 VTE cases with therapeutic anticoagulation evidence, 29 (67.4%) did not have AF, suggesting that the majority of therapeutic anticoagulation was prescribed for VTE rather than AF management.

Due to the small number of high-confidence VTE events (*n* = 4), multivariable logistic regression analysis restricted to this subgroup was not performed, as such analyses would not yield reliable estimates.

### Interaction analysis

Exploratory interaction analyses revealed no significant multiplicative interaction between AF and mechanical ventilation on VTE risk (interaction OR = 1.87, 95% CI: 0.39–9.06, *p* = 0.436; likelihood ratio test *p* = 0.431). The AF × COPD interaction could not be reliably estimated due to sparse data in the combined subgroup (AF = 1 and COPD = 1: *n* = 5, of whom 3 developed VTE), resulting in quasi-complete separation and model instability. Detailed interaction analysis results are presented in Supplementary Table 4.

### AF subgroup characteristics

Baseline characteristics stratified by AF status are presented in Supplementary Table 5. Compared with patients without AF, AF patients were significantly older (70.7 ± 17.2 vs. 59.3 ± 20.2 years, *p* < 0.001), had higher APACHE IV scores (63.7 ± 27.8 vs. 54.8 ± 27.1, *p* = 0.009), and had greater prevalence of hypertension (41.9% vs. 19.8%, p < 0.001), congestive heart failure (6.5% vs. 0.8%, *p* = 0.005), and COPD (12.9% vs. 2.1%, p < 0.001). SCD use was lower in AF patients (77.4% vs. 87.4%, *p* = 0.033). AF patients also had longer ICU stays (6.1 ± 7.8 vs. 4.2 ± 7.8 days, *p* = 0.001) and hospital stays (12.5 ± 11.5 vs. 8.6 ± 9.0 days, *p* = 0.001).

## Discussion

In this multicenter retrospective cohort study of 785 patients with ICH, atrial fibrillation was the only independent risk factor for VTE after multivariable adjustment for disease severity, comorbidities, and thromboprophylaxis measures, and should be interpreted as a clinical risk marker rather than a direct causal factor. Patients with AF exhibited a markedly higher incidence of VTE compared with those without AF (24.2% vs. 5.3%), representing an unadjusted 4.6-fold difference in VTE incidence. After multivariable adjustment, AF conferred an approximately four-fold increase in VTE risk (adjusted OR = 3.84, 95% CI: 1.75–8.41, *p* < 0.001). This association remained consistent across different VTE definitions, including a sensitivity analysis excluding anticoagulation-only VTE cases, supporting the robustness and clinical relevance of the primary findings.

The overall VTE incidence observed in our cohort (6.75%) is consistent with rates reported in previous studies of ICH, which have ranged from approximately 1 to 10% depending on study design and case ascertainment methods (3, 5, 6, 8, 11, 14, 15). Earlier investigations relying primarily on administrative coding or small cohorts tended to report lower incidence rates, whereas studies incorporating more detailed clinical assessment identified higher rates of VTE. In the present study, VTE events were identified using a multi-source evidence integration strategy that combined diagnostic information and therapeutic anticoagulation records, with events further stratified by confidence level. This approach was intended to enhance sensitivity while maintaining transparency in case classification (Supplementary Table 1).

Several prior studies have reported associations between VTE and clinical factors such as lower extremity weakness, immobility, advanced age, mechanical ventilation, and sex-specific differences in patients with ICH (6, 8, 10, 11, 15–17). However, these studies were generally limited by small sample sizes, heterogeneous adjustment strategies, and incomplete control for overall disease severity. Notably, AF has not been consistently identified as an independent VTE risk factor in previous ICH studies, likely reflecting differences in sample size, AF prevalence, and statistical adjustment strategies. A structured comparison of prior literature demonstrates that AF was not systematically evaluated as a candidate risk factor in earlier ICH cohorts (Supplementary Table 2; expanded to include recent studies by Christensen et al. (18), Diao et al. (19), and Salvagni et al. (20)).

The discrepancy in AF’s identification as a VTE predictor across studies likely reflects several methodological differences. First, sample size and event rates-many prior studies had limited power to detect moderate associations (EPV < 10 for AF). Second, VTE ascertainment methods-studies relying solely on ICD codes may miss clinically significant VTE events, while those employing systematic screening detect higher incidence. Third, population heterogeneity-studies including both surgical and non-surgical ICH, or mixing ICU and ward patients, may dilute subgroup-specific effects. Fourth, covariate adjustment-the specific variables included in multivariable models varied substantially across studies, affecting whether AF emerged as an independent predictor after adjustment.

In contrast, the present study incorporated validated measures of disease severity, including APACHE IV and GCS scores, and applied multivariable modeling in a cohort of adequate size. Although several variables showed significant associations with VTE in univariate analyses, only AF retained independent significance after adjustment. This finding suggests that previously reported risk factors may, at least in part, represent surrogate markers of overall illness severity rather than independent predictors of venous thrombosis.

The strong association between AF and VTE observed in this study represents a novel contribution to the existing literature. While AF is well established as a risk factor for arterial thromboembolism, its role in venous thrombosis has been less clearly defined.

### Mechanisms of AF-ICH synergistic VTE risk

Several synergistic mechanisms may explain the elevated VTE risk in ICH patients with AF:

First, acute brain injury-related coagulopathy-ICH triggers massive tissue factor release and sympathetic hyperactivation, leading to a systemic hypercoagulable state through enhanced thrombin generation and impaired fibrinolysis (21, 22). When superimposed on the chronic prothrombotic milieu of AF (including elevated von Willebrand factor, P-selectin, and D-dimer levels), the combined effect may substantially amplify thrombotic risk.

Second, prolonged immobilization-AF patients in our cohort had significantly longer ICU stays (6.1 vs. 4.2 days, *p* = 0.001) and hospital stays (12.5 vs. 8.6 days, *p* = 0.001), as well as higher APACHE scores (63.7 vs. 54.8, *p* = 0.009), suggesting greater illness severity. Extended immobilization promotes venous stasis through reduced calf muscle pump function, a well-established contributor to DVT pathogenesis (Virchow’s triad) (17).

Third, endothelial dysfunction-Both AF and acute brain injury independently activate the endothelium through inflammatory mediators (IL-6, TNF-alpha), complement activation, and neutrophil extracellular trap (NET) formation (23, 24). The convergence of these pathways may create a permissive vascular environment for thrombus formation.

Fourth, autonomic dysfunction-ICH-induced sympathetic surge increases heart rate, blood pressure variability, and platelet activation, which may be exacerbated by the irregular hemodynamic patterns inherent to AF (25).

### Anticoagulant withdrawal rebound

An important clinical consideration is the potential prothrombotic rebound effect following anticoagulant discontinuation in AF patients. Many AF patients receive long-term oral anticoagulation (warfarin or DOACs) for stroke prevention, which must be withheld after ICH onset due to bleeding concerns. Abrupt anticoagulant withdrawal has been associated with a transient hypercoagulable state characterized by increased thrombin generation, elevated D-dimer, and reduced protein C and S levels (26, 27). In the context of ICH-induced immobilization and acute inflammatory response, this rebound hypercoagulability may serve as a potent VTE trigger. Unfortunately, the eICU database does not capture pre-admission medication histories, precluding direct analysis of this mechanism. Future studies should specifically examine the temporal relationship between anticoagulant cessation and VTE occurrence in ICH patients with AF.

### COPD effect attenuation

The substantial attenuation of the COPD effect (unadjusted OR = 6.81 to adjusted OR = 2.30, *p* = 0.250; a 66% reduction) warrants discussion. Our analysis reveals that this is largely explained by the strong confounding between COPD and AF: 34.8% of COPD patients had concurrent AF, compared to only 7.1% of non-COPD patients (OR = 6.99, *p* < 0.001). When COPD was adjusted for AF alone (without other covariates), its OR decreased from 6.81 to 4.40, confirming that approximately 35% of the COPD effect was mediated through or confounded by AF. Three mechanisms explain this confounding: (1) COPD and AF share common pathophysiological pathways including chronic systemic inflammation, hypoxia-induced atrial remodeling, and pulmonary hypertension (28); (2) the COPD-AF comorbidity is epidemiologically well-established (prevalence of AF in COPD: 15–25%); and (3) with only 23 COPD patients in our cohort (EPV = 2.3), statistical power was insufficient to detect an independent COPD effect after adjustment. Future studies with larger COPD subgroups should investigate whether COPD confers additional VTE risk beyond that mediated by AF.

### AF as risk marker vs. causal agent

We recognize that the observed AF-VTE association may partially reflect an indirect causal pathway: AF patients are more likely to have been on chronic anticoagulation before ICH, which may lead to more severe hemorrhage, greater neurological impairment, longer immobilization, and consequently higher VTE risk. Although we adjusted for GCS (as a proxy for neurological severity) and APACHE score, we lacked direct measures of hematoma severity, ICH volume, and pre-admission anticoagulation status. Therefore, the AF coefficient in our model likely captures both the direct prothrombotic effect of AF and the indirect effect mediated through ICH severity and immobilization. Future studies with detailed neuroimaging data and pre-admission medication records are needed to disentangle these pathways. We caution that AF should be interpreted as a risk marker associated with a constellation of VTE-promoting factors, rather than necessarily a direct causal agent.

### VTE outcome misclassification

A key methodological consideration is the potential for outcome misclassification when using therapeutic anticoagulation as evidence of VTE. In patients with AF, therapeutic anticoagulation may reflect AF management rather than VTE treatment, creating differential misclassification that could bias the AF-VTE association. However, several observations mitigate this concern. First, our sensitivity analysis excluding anticoagulation-only VTE cases confirmed the association (adjusted OR = 3.99, *p* = 0.046). Second, among the 43 anticoagulation-associated VTE cases, 67.4% occurred in non-AF patients, suggesting that VTE was the primary indication in most cases. Third, in the eICU setting, AF management typically involves rate or rhythm control agents rather than therapeutic anticoagulation during the acute ICH phase, as anticoagulation is contraindicated. Nevertheless, we cannot completely exclude misclassification, and this represents an important limitation.

From a clinical perspective, these findings have important implications for risk stratification and thromboprophylaxis in ICH. Previous approaches have largely emphasized neurological deficits and immobility as markers of VTE risk (8, 10, 15), whereas cardiac rhythm disorders have received limited attention. Identification of AF as a strong and independent risk marker may facilitate earlier recognition of high-risk patients and support more individualized monitoring and prevention strategies. Notably, SCD use was lower in AF patients (77.4% vs. 87.4%, *p* = 0.033), which may itself contribute to higher VTE risk and suggests an actionable target for quality improvement.

## Limitations

### Several limitations of this study should be acknowledged

First, the large exclusion rate (73.6%) raises concerns about selection bias. The majority of exclusions were due to missing APACHE IV scores, which require complete physiological data within the first 24 h of ICU admission. Patients with very short ICU stays, rapid deterioration, or transfers may be underrepresented, potentially limiting generalizability to the broader ICH population. The final cohort may overrepresent patients with more stable clinical courses who remained in ICU long enough for complete data capture.

Second, the retrospective observational design precludes causal inference, and residual confounding cannot be fully excluded. Important clinical variables such as hemorrhage volume, anatomical location, surgical interventions, pre-ICH anticoagulation use, and NIHSS scores were not available. Although we adjusted for GCS and APACHE score, these are imperfect surrogates for neurological severity and ICH characteristics.

Third, certain VTE risk factors including prior VTE history and inherited thrombophilia were not available in the database, which may contribute to residual confounding.

Fourth, the eICU database captures only ICU-admitted patients, representing a more severely affected subset of all ICH patients. Milder ICH cases managed on general wards or discharged early from the emergency department are not represented. Our findings therefore apply specifically to ICH patients requiring ICU-level care and may not generalize to the full spectrum of ICH severity. The VTE incidence (6.75%) in our cohort is within the range reported for ICU-based studies (5–12%) but may overestimate the risk in the general ICH population.

Fifth, VTE identification relied on a multi-source approach combining ICD-9 codes, text mining, and therapeutic anticoagulation records, rather than imaging confirmation. Although sensitivity analyses excluding anticoagulation-only cases confirmed the robustness of the AF-VTE association, outcome misclassification remains possible, particularly because therapeutic anticoagulation in AF patients may reflect AF management rather than VTE treatment.

Sixth, the eICU database does not provide sufficient detail to classify DVT by anatomical location (proximal vs. distal). This distinction is clinically important, as proximal DVT carries higher risk of pulmonary embolism and typically requires different management strategies.

Seventh, the AF variable in our study reflects pre-existing AF documented at ICU admission and may not capture new-onset AF during the ICU stay. The eICU database does not provide sufficient detail to classify AF subtypes (paroxysmal, persistent, or permanent), AF duration, or prior anticoagulation regimens. Different AF subtypes may carry different thrombotic risk profiles, and this inability to differentiate represents an important limitation.

Eighth, the eICU data was collected during 2014–2015, a period when DOACs were increasingly used for AF stroke prevention but were not yet the dominant anticoagulation strategy. In the current era of widespread DOAC use, the prevalence of prior anticoagulation among AF patients with ICH may be higher, and the clinical characteristics of DOAC-associated ICH may differ from warfarin-associated ICH. However, the fundamental pathophysiological relationship between AF and VTE is unlikely to be era-dependent. Validation in contemporary cohorts is warranted.

Future studies are warranted to validate these findings in prospective and external cohorts, to further elucidate the biological mechanisms linking AF and venous thrombosis after ICH, and to determine optimal prophylactic and anticoagulation strategies in this high-risk population (29). Studies with larger sample sizes should also explore potential synergistic effects between AF and other covariates, particularly COPD, which could not be adequately assessed in our cohort.

## Conclusion

Atrial fibrillation was the only independent risk factor for VTE in patients with ICH after multivariable adjustment, conferring an approximately fourfold increase in risk (adjusted OR = 3.84, 95% CI: 1.75–8.41, *p* < 0.001). Patients with ICH and concomitant AF constitute a high-risk subgroup, with VTE incidence of 24.2%. AF should be interpreted as a clinical risk marker rather than a direct causal factor. These findings have important implications for clinical risk stratification and support the need for enhanced surveillance and individualized thromboprophylaxis strategies in this population. Given that the use of pharmacological anticoagulation after ICH requires careful risk–benefit assessment due to the presence of recent intracranial hemorrhage, aggressive mechanical prophylaxis may be particularly warranted. Future prospective studies are needed to validate these results and to define optimal VTE prevention strategies in this vulnerable group.

## Data Availability

The datasets presented in this study can be found in online repositories. The names of the repository/repositories and accession number(s) can be found in the article/Supplementary material.
