# Supplementary material for: Atrial fibrillation as an independent risk factor for venous thromboembolism in intracerebral hemorrhage patients: a multicenter retrospective cohort study
Source: Front Neurol. 2026 May 22;17:1796208. doi: 10.3389/fneur.2026.1796208 (PMC13238432; doi:10.3389/fneur.2026.1796208)
Supplement: Supplementary file 2 [file Supplementary_file_1.pdf]

Supplementary Table 1. VTE Evidence Sources and Confidence Classification

Evidence Source	Number of Patients	Percentage (%)
**Diagnostic Evidence**		
DVT — ICD-9 code	0	0%
DVT — Text mining	33	4.2%
PE — ICD-9 code	0	0%
PE — Text mining	5	0.6%
Other thrombosis diagnosis	3	0.4%
**Therapeutic Evidence**		
Warfarin	52	6.6%
Therapeutic-dose LMWH	77	9.8%
Heparin infusion	23	2.9%
DOAC	0	0%
**Confidence Categories**		
High confidence (Diagnosis + Treatment)	4	0.5%
Medium confidence (Diagnosis OR Treatment)	49	6.2%
Low confidence (Weak evidence)	0	0%
**Total VTE (Recommended)**	**53**	**6.8%**

Abbreviations: VTE, venous thromboembolism; DVT, deep vein thrombosis; ICD-9, International Classification of Diseases, Ninth Revision; PE, pulmonary embolism; LMWH, low-molecular-weight heparin; DOAC, direct oral anticoagulant.

Note: Patients may have multiple sources of evidence; percentages calculated based on total cohort of 785 patients.

Supplementary Table 2. Comparison with Previous Literature [REVISED]

Study	Year	Population	N	VTE Incidence	Key Risk Factors	AF Evaluated?
Gregory et al. [10]	2003	ICH and ischemic stroke	98 ICH	2.0%	Lower extremity weakness	No
Skaf et al. [6]	2005	ICH and ischemic stroke	14,953	0.6%	Age, prior VTE	No
Lacut et al. [3]	2005	ICH	226	2.2%	Lower limb motor deficit	No
Ogata et al. [15]	2008	ICH	97	7.2%	Leg paresis, bedridden	No
Kawase et al. [11]	2009	ICH	168	4.8%	Female sex, lower GCS	No
Goldstein et al. [8]	2009	ICH	155	7.7%	Immobility, mech vent	No
Christensen et al. [18]	2019	ICH (ESO guideline)	14,572	—	Anticoagulation reversal in AF-related ICH (qualitative guideline)	Yes
Salvagni FP et al. [20]	2024	ICH (meta-analysis)	—	—	VTE prophylaxis timing – early vs late	Yes (qualitative)
Diao H et al. [19]	2024	ICH (meta-analysis, multiple cohorts)	18 studies	—	Multiple factors including AF (qualitative)	Yes
Current Study	2024	ICH (multicenter ICU)	785	6.8%	AF (only independent)	Yes

Abbreviations: ICH, intracerebral hemorrhage; VTE, venous thromboembolism; AF, atrial fibrillation; GCS, Glasgow Coma Scale; HR, hazard ratio; OR, odds ratio.

Supplementary Table 3. Comparison of Included vs. Excluded Patients [REVISED]

Variable	Included (n = 785)	Excluded (n = 2,193)	P value
**Exclusion Reasons**			
Missing APACHE IV score	—	1,253 (57.1%)	—
Missing age/sex	—	584 (26.6%)	—
Data quality issues	—	356 (16.2%)	—

Note: Detailed comparison is limited by the nature of the exclusion criteria. Patients excluded for missing APACHE IV scores lacked complete first-24-hour physiological data, likely reflecting very short ICU stays (<24 hours), early transfers, or technical issues with data capture. The final cohort may overrepresent patients with more stable clinical courses who remained in ICU long enough for complete data capture.

Supplementary Table 4. Interaction Analysis Results [REVISED]

Interaction Term	Interaction OR	95% CI	Wald P value	LR Test P value	Interpretation
AF × Mechanical Ventilation	1.87	0.39–9.06	0.436	0.431	No significant interaction
AF × COPD	Not estimable	—	—	—	Sparse data[a]

[a] The AF = 1 / COPD = 1 subgroup contained only 5 patients (3 with VTE), resulting in quasi-complete separation and model instability.

Base model: VTE ~ AF + COPD + Mechanical Ventilation + SCD use + GCS score (n = 725 complete cases, 45 VTE events).

Abbreviations: AF, atrial fibrillation; COPD, chronic obstructive pulmonary disease; OR, odds ratio; CI, confidence interval; LR, likelihood ratio; SCD, sequential compression device; GCS, Glasgow Coma Scale.

Supplementary Table 5. Baseline Characteristics Stratified by Atrial Fibrillation Status [REVISED]

Variable	AF (n = 62)	No AF (n = 723)	P value
**Demographics**			
Age, years (mean ± SD)[b]	70.7 ± 17.2	59.3 ± 20.2	<0.001***
Male, n (%)[b]	20 (35.7%)	286 (42.9%)	0.367
**Disease Severity**			
APACHE IV score (mean ± SD)[b]	63.7 ± 27.8	54.8 ± 27.1	0.009**
GCS score (mean ± SD)[b]	10.3 ± 5.1	10.7 ± 4.8	0.491
Mechanical ventilation, n (%)[b]	20 (35.7%)	202 (30.2%)	0.478
**Comorbidities**			
Hypertension, n (%)	26 (41.9%)	143 (19.8%)	<0.001***
Diabetes mellitus, n (%)	1 (1.6%)	7 (1.0%)	0.484
Chronic kidney disease, n (%)	5 (8.1%)	23 (3.2%)	0.062
Congestive heart failure, n (%)	4 (6.5%)	6 (0.8%)	0.005**
COPD, n (%)	8 (12.9%)	15 (2.1%)	<0.001***
**Laboratory Values**			
Hemoglobin, g/dL (mean ± SD)[a,b]	12.1 ± 2.5	12.5 ± 2.7	0.082
**Thromboprophylaxis**			
SCD use, n (%)	48 (77.4%)	632 (87.4%)	0.033*
**Outcomes**			
VTE events, n (%)	15 (24.2%)	38 (5.3%)	<0.001***
Therapeutic anticoagulation, n (%)	14 (22.6%)	29 (4.0%)	<0.001***
ICU LOS, days (mean ± SD)[b]	6.1 ± 7.8	4.2 ± 7.8	0.001**
Hospital LOS, days (mean ± SD)[b]	12.5 ± 11.5	8.6 ± 9.0	0.001**

[a] After removal of implausible outliers (>30 g/dL).

[b] Variables with missing data: sex and mechanical ventilation unavailable for 6 AF and 54 non-AF patients; age for 7 AF and 77 non-AF; APACHE IV for 12 AF and 113 non-AF; GCS for 6 AF and 54 non-AF; ICU/Hospital LOS for 12 AF and 113 non-AF patients. Percentages calculated using available-case denominators. Continuous variables compared using Mann-Whitney U test; categorical variables using chi-square or Fisher's exact test (expected cell count <5).

Note: The eICU database does not provide sufficient detail to classify AF subtypes (paroxysmal, persistent, or permanent), AF duration, or prior anticoagulation regimens.
Abbreviations: AF, atrial fibrillation; SD, standard deviation; APACHE, Acute Physiology and Chronic Health Evaluation; GCS, Glasgow Coma Scale; COPD, chronic obstructive pulmonary disease; SCD, sequential compression device; VTE, venous thromboembolism; ICU, intensive care unit; LOS, length of stay.
*P < 0.05; **P < 0.01; ***P < 0.001
